# SSR Genotyping and Marker–Trait Association with Yield Components in a Kazakh Germplasm Collection of Chickpea (*Cicer arietinum* L.)

**DOI:** 10.3390/biom13121722

**Published:** 2023-11-29

**Authors:** Shynar Mazkirat, Kuralay Baitarakova, Mukhtar Kudaybergenov, Dilyara Babissekova, Sholpan Bastaubayeva, Kulpash Bulatova, Yuri Shavrukov

**Affiliations:** 1 Kazakh Research Institute of Agriculture and Plant Growing, Almaty District, Almalybak 040909, Kazakhstan; kuralai_baitarakova@mail.ru (K.B.); muhtar.sarsenbek@mail.ru (M.K.); janeka__88@mail.ru (D.B.); sh.bastaubaeva@mail.ru (S.B.); bulatova_k@rambler.ru (K.B.); 2 College of Science and Engineering, Biological Sciences, Flinders University, Adelaide, SA 5042, Australia

**Keywords:** autumn and spring sowing, chickpea germplasm collection, marker–trait association, molecular markers, SSR genotyping, yield components

## Abstract

Genetic diversity and marker–trait association with yield-related components were assessed in 39 chickpea accessions from a germplasm collection with either spring or autumn-sown seeds in South-Eastern Kazakhstan. Chickpea accessions originated from Azerbaijan, Germany, Kazakhstan, Moldova, Russia, Türkiye, Ukraine, Syria, and the International Center for Agricultural Research in the Dry Areas (ICARDA). Eleven SSR markers were used for molecular genotyping. Yield and yield components were evaluated in nine traits in experiments with spring and autumn seed sowing. The number of alleles of polymorphic markers varied from 2 to 11. The greatest polymorphism was found in the studied chickpea genotypes using SSR marker TA22 (11 alleles), while NCPGR6 and NCPGR12 markers were monomorphic. In the studied chickpea accessions, unique alleles of the SSR loci TA14, TA46, TA76s, and TA142 were found that were not previously described by other authors. An analysis of correlation relationships between yield-related traits in chickpea revealed the dependence of yield on plant height, branching, and the setting of a large number of beans. These traits showed maximal values in experiments with chickpea plants from autumn seed sowing. An analysis of the relationship between the SSR markers applied and morphological yield-related traits revealed several informative markers associated with important traits, such as plant height, height to first pod, number of branches, number of productive nodes, number of pods per plant, hundred seed weight, seed weight per plant, and seed yield.

## 1. Introduction

Chickpea (*Cicer arietinum* L.) is an annual, self-pollinated diploid legume plant species, from the Fabaceae family, providing high-quality plant protein for more than 30% of the world’s population. In 2018, 17.2 million tons of chickpeas were produced from 17.8 million hectares of land throughout the world [1]. In Kazakhstan, the area used for chickpea growing has consistently increased due to its high profitability and the soil fertility improvements achieved through atmospheric nitrogen fixation. The area of chickpea crop production in Kazakhstan currently covers more than 23,000 hectares [2], and Northern and Central regions of the country use chickpea as a spring-sown crop [3,4].

The ability of chickpea plants to withstand drought and moisture deficiency is a key to its successful cultivation in non-irrigated conditions of Kazakhstan [5,6]. Additionally, chickpea plants can be tolerant to cold stress from low temperatures and even a short time of freezing. Autumn-sown chickpea, therefore, is also used in Southern and South-Eastern Kazakhstan where there are mild-cold winters, and plants grow and overwinter in these conditions [7].

The effects of global climate warming on crop growth and land use are impossible to ignore. In Southern Kazakhstan, farmers increasingly prefer chickpea cultivars suitable for autumn sowing. The reproductive phase of chickpea growth, including flowering, pod setting, and seed maturation, represent the most critical periods of ontogenesis and development. With spring sowing, chickpea plants risk more frequent and often overlapping periods where reproductive organs are exposed to severe drought, resulting in significantly reduced seed yields. In contrast, chickpea plants after autumn sowing can start growth earlier and avoid the drought period, thereby minimizing the risk of damage [8,9,10,11]. Autumn sowing is recommended to obtain high yields since plants can exploit the available spring moisture (melting snow and precipitation), to extend their development period, resulting in a broader canopy and overall larger plant habit, which has a positive effect on their photosynthetic ability [12,13,14].

Mild winter conditions in Mediterranean environments can also provide the opportunity to sow chickpea seeds in autumn. This was shown to increase yield and yield stability, profiting from winter rains and minimizing the effects of terminal heat and drought stress [15]. Such changes in the sowing season can significantly improve chickpea plant growth and seed yield production in Kazakhstan.

In Uzbekistan, a country close to Kazakhstan, cold-tolerant chickpea cultivars are often sown in autumn if winter temperatures do not fall below −10 °C for a few days. Autumn rains and melting snow can increase soil fertility, providing a rapid start of chickpea plant growth in spring and avoiding heat and drought during flowering and subsequent pod and seed development. As a result, three new chickpea cvs., Abad, Malhotra, and Khalima, were released in Uzbekistan that were suitable for planting in autumn [16].

It is important to note that autumn-sown chickpea yields can be comparable with those of the spring-sown type with the development of cold-tolerant chickpea cultivars with increased productivity [17]. Screening against low temperatures has been taken up vigorously in recent years. In Iran, for example, cold-tolerant chickpea genotypes were identified and introduced during field and controlled environment screening [18].

The genetic diversity germplasm collections of crops including chickpea can be an essential resource for the identification and use of required genotypes adapted to specific regional growing conditions, resulting in their successful application in breeding [19,20].

Applying molecular markers in genetic diversity studies can enable germplasm collections to be used more effectively [21]. The discovery of molecular markers closely linked to the studied breeding traits has made it possible to carry out marker-assisted selection, MAS [22]. In the breeding of chickpea and other legume crops, microsatellite markers, SSR, have been successfully used for the assessment of genetic diversity and further selection, including MAS [23,24,25,26,27,28,29].

SSR markers have a codominant inheritance type, which is very important for hybrid population analyses [30]. A very high polymorphism of SSR loci and allele distribution throughout the genome provides very diverse information about the genetic polymorphism among studied germplasms [31,32,33,34,35,36].

The reliable, highly reproducible, and low-cost nature of genotyping assays using SSR markers make it one of the simplest and most suitable molecular tools for MAS in many crops [37,38]. Simple codominant inheritance, reproducibility, bi- or multi-allelic nature, and abundant genomic distribution represent desirable genetic attributes of sequence-based robust SSR markers [39]. Therefore, SSRs are used in many applications of chickpea genetics, genomics, and breeding, including cultivar identification, allele mining, genetic mapping, association studies, genetic diversity analysis, population structure studies, and the establishment of phylogenetic relationships [19,40].

The purposes of this research were (1) to assess the genetic diversity in a chickpea germplasm collection in Kazakhstan using SSR markers; and (2) to analyze associations between SSR allelic variants and yield components in chickpea genotypes for better productivity in plants grown in autumn and spring sowing periods.

## 2. Materials and Methods

### 2.1. Plant Material and Field Experiments

In this study, 39 chickpea accessions were used from the legume germplasm collection of the Kazakh Research Institute of Agriculture and Plant Growing (KRIAPG), Almaty region, Kazakhstan (Table 1). The germplasm collection included both cultivars and breeding lines from Azerbaijan, Germany, Kazakhstan, Moldova, Russia, Türkiye, Ukraine, Syria, and the International Center for Agricultural Research in the Dry Areas (ICARDA). Morphological characteristics of seeds and plants were determined in accordance with the recommendations of the International Union for the Protection of New Varieties of Plants Guidelines, and digital notes (score numbers) were indicated for studied traits [41]. All studied chickpea accessions were of the Kabuli ecotype with bigger and light-colored seeds, and only one exception of the cv. Malhotra, of the Desi ecotype, with smaller and darker-colored seeds.

Chickpea germplasm accessions were assessed during spring and autumn sowing in 2016 and 2017 in research fields of KRIAPG, located in South-Eastern Kazakhstan, at 43° N. The climatic condition of 2016 was more favorable and closer to the average for many years while 2017 had an almost 6 °C higher temperature balance, especially in spring season. In general, the climate in the region is continental with hot summers and humid winters, and estimated as ‘Dfa’, according to the Köppen classification [42]. The average temperature in January is about −6 to −9 °C, while in July it is +22 to +24 °C, and annual precipitation ranges from 300 to 500 mm [43].

Seed sowing was carried out manually in a plot area measuring 1 m^2^, and 60 seeds for each accession in each plot were sown in a completely randomized block design with three replicates, with 180 grown plants for each accession in total. The chickpea domestic cv. Kamila was used as a standard (check) for the comparisons. Ten randomly selected plants per accession were assessed for the following nine traits: (1) plant height (PH), the length of the longest stem from soil (cm); (2) height to first pod (HFP), the distance from the soil at the base of the plant to the first pod (cm); (3) number of branches (NB), counted as the number of branches in the longest stem; (4) number of productive nodes (NPN), counted as the number of nodes on the longest stem with pods; (5) number of pods per plant (NPP), counted as the number of pods in each plant; (6) seed weight per plant (SWP), or the weight of all cleaned seeds produced in each plant measured on a laboratory scale (g); (7) 100 seed weight (HSW), the weight of 100 seeds measured on a laboratory scale (g); (8) yield (Yd), calculated as seed yield (tons/hectare) based on SWP × number of plants per plot × coefficient from the equation [1 t/ha = 0.1 kg/m^2^] (t/ha); and (9) vegetation period length (VP), the number of days from seed sowing to full maturation at harvesting (days). The measurement and calculation of the studied traits were in accordance with the methodological guidelines [44].

### 2.2. Molecular Analysis

DNA was extracted using the CTAB method [45]. Briefly, 200 mg of leaf tissue collected from five plants for each accession was ground to a powder using a pre-cooled mortar and pestle, and then transferred into 2 mL test tubes with 600 µL of extraction buffer. DNA was precipitated using pre-cooled 100% ethanol and washed with 70% ethanol. DNA was dissolved with ddH_2_O. The dissolved DNA was treated with 1 µL of RNase A, at 10 µg/µL (Thermo Fisher Scientific, Waltham, MA, USA) and 37 °C for 30 min. The quality and quantity of the extracted DNA was determined by measuring absorbance at 260 and 280 nm using a Jenway 635031 spectrophotometer (Bibby Scientific, Ltd., Staffordshire, UK). DNA concentration was diluted to 100 ng/µL using ddH_2_O.

PCR was carried out in a total volume of 15 μL containing 1.5 μL of 10 × PCR buffer, 2 mM MgCl_2_, 0.15 μM of each dNTP, 0.7 μM of each primer, 0.5 U of Taq DNA polymerase (GeneLab, Astana, Kazakhstan) and 0.07 μg/μL BSA (final concentration). The reactions were carried out in a Bio-Rad iCycler system (Bio-Rad Laboratories, Hercules, CA, USA) with the following program: an initial step of 94 °C for 3 min; 30 cycles of 94 °C for 1 min; 55 °C for 1 min and 72 °C for 1 min; and a final step of 72 °C for 5 min. PCR products were visualized on 8% polyacrylamide gel at a constant rate of 200 V for 70 min and detected via staining with ethidium bromide. Electrophoresis images were obtained using a Quantum ST4 gel-documenting system (Vilber, Lourmat, Collégien, France). The amplicon size was determined using the computer program Quantum Capture Image Analysis (Vilber, Lourmat, Collégien, France).

To assess the genetic diversity of the studied chickpea genotypes, 11 SSR markers were used (Table 2) that were selected from 35 SSR markers previously used and recommended for studying chickpea genetic diversity [36]. Descriptive details of the amplification products for alleles of SSR loci are provided in Supplementary Material S1.

### 2.3. Data Analysis

For scoring each polymorphic band, the patterns of SSR loci were evaluated using the following codes: ‘1’ for the presence of the band and ‘0’ for band absence. A data matrix for bands’ presence or absence was used for further analysis. The number of alleles [46], major allele frequency, genetic diversity [47], and polymorphism information content (PIC) [48] were calculated using PowerMarker 3.25 software [49]. Cluster analysis using the unweighted pair group method with arithmetic mean (UPGMA) and Pearson’s correlation analysis was conducted in R software (v4.1.3) [50].

STRUCTURE v2.3.4 program software was used to analyze the structure of the populations using a Bayesian–Markov chain–Monte Carlo (MCMC) technique based on the admixture and correlated allele frequency [51]. The data set was run through 10,000 Markov chain–Monte Carlo iterations with an initial burn-in period of 10,000 with five replicates, considering several subgroups (K) ranging from 1 to 10. Structure Harvester was used to determine the optimal k-value (http://taylor0.biology.ucla.edu/structureHarvester, accessed on 2 October 2023) as well as to illustrate the results obtained from STRUCTURE v2.3.4.

The marker–trait association analysis between the SSR markers and morphological traits was performed using the mixed linear model (MLM) with a kinship matrix and Q-matrix in TASSEL 5.0. MLM (K+Q), which can reduce type I errors in contrast to the general linear model (GLM), and prevent more false positives. Markers with a minimum frequency of alleles of less than 5% were excluded and the kinship matrix was obtained based on all SSR markers using TASSEL 5.0 [52].

The legume information system (LIS) was used for searching candidate genes and their location in the annotated genomes of the chickpea cv. Frontier using the LegumeMine database [53].

## 3. Results

### 3.1. Genetic Diversity of Chickpea Germplasm Collection

**Figure 1:**
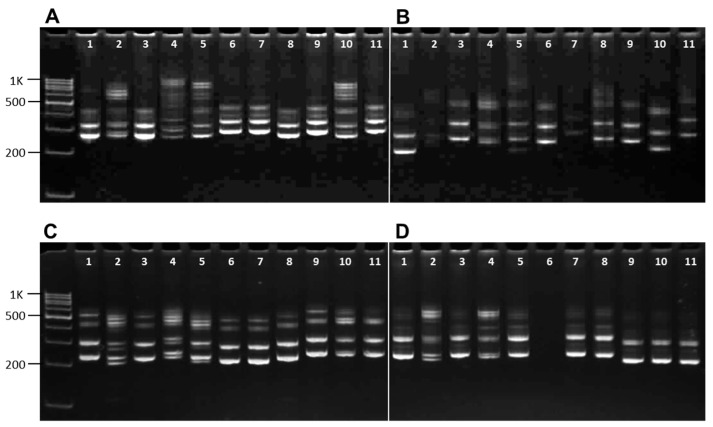
The example of genetic polymorphism identification in molecular markers with band separation on 8% polyacrylamide gel without any adjustment. SSR amplification products from (**A**) TA14; (**B**) TA22; (**C**) TA71; and (**D**) TA76s. Eleven used samples are indicated by numbers with corresponding IDs in Table 1: (1) 33, Kamila; (2) 36, Luch; (3) 5, 30112; (4) 39, Vysokoroslyj; (5) 19, Ezbsen Sponishe; (6) 15, 28-B; (7) 37, Malhotra; (8) 18, 34-B; (9) 34, Liniya-7B; (10) 31, F98-130; and (11) 38, S-35. Sizes of a 100 bp DNA ladder are present and indicated on the left-hand side.

Alleles of SSR markers identified in the screening of the chickpea germplasm collection were studied and used in the breeding programs at KRIAPG. The example of SSR markers band separation on polyacrylamide gel is shown in Figure 1.

In the studied chickpea accessions, some alleles for the SSR loci TA14, TA46, TA76s, and TA142 were identified for the first time in this study and were not previously described or reported. Interestingly, new alleles identified in our study tended to have larger amplicon sizes. For example, for the TA14 marker, new alleles were found with amplicons for 288, 300, and 307 bp, while only 267 and 278 bp products were known earlier. Other SSR markers with novel alleles included TA46, TA76s, and TA142. Additionally, marker TA142 can be estimated as the most polymorphic for all identified alleles among all studied chickpea accessions. In contrast, markers NCPGR6 and NCPGR12 were represented only by monomorphic spectra and thus were excluded from further analyses (Table 3).

In total, 51 alleles were revealed among all SSR markers used. The observed number of alleles ranged from 2 (NCPGR19) to 11 (TA22). All SSR markers showed high Nei’s genetic diversity ranging from 0.46 (TA76s) to 0.87 (TA22), and the average genetic diversity for all loci was 0.69. This indicated high allele diversity among the selected SSR markers in this study (Table 4).

All used SSR loci in this study had high PIC values with an average of 0.65. The marker TA22 showed the highest value, PIC = 0.86, whereas the marker NCPGR19 had the lowest PIC value of 0.36. Marker TA22 produced 11 alleles with the highest gene diversity and PIC values. According to the overview results in this study for chickpea gene diversity, primers TA14, TA22, TA46, TA71, and NCPGR4 could be the most effective loci among the nine microsatellite primers used in this study.

### 3.2. Cluster Analysis

Various algorithms and tools, such as the unweighted pair group method with arithmetic mean (UPGMA), a Bayesian approach (STRUCTURE analysis), etc., have been used on genetic clustering depending on the study design and marker types. In the current study, UPGMA was used to differentiate accessions based on their genetic distance. Furthermore, a Bayesian approach (STRUCTURE analysis) was also used to infer genetic structure and validate and compare the results obtained using UPGMA methods.

**Figure 2:**
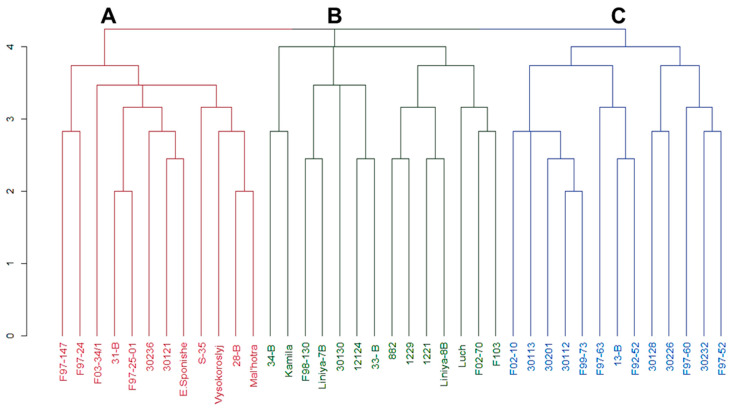
Dendrogram derived from UPGMA cluster analysis of nine SSR marker alleles of 39 chickpea accessions distributed in three clusters (A–C). The names of the accessions used are indicated in the x-axis, while the y-axis corresponds to the linkage distances between clusters.

The genetic diversity of the studied chickpea germplasm collection was assessed via a cluster analysis of SSRs. The results showed a precise distribution of studied genotypes among three clusters according to the allelic composition of the analyzed markers (Figure 2). The first cluster, Cluster A, contained 12 samples, including samples with specific alleles of the TA76s and NCPGR7 markers with band sizes of 230 and 214 bp, respectively. Among the genotypes in Cluster A were chickpea accessions predominantly from Kazakhstan. It should also be noted that the cv. Malhotra (of the Desi ecotype) was also among this group of accessions in Cluster A.

Cluster B, with 14 accessions, had a significant part (86%) formed by accessions with specific alleles of the NCPGR7 and TA142 markers, with 211 and 147 bp band sizes, respectively. This cluster also consisted of chickpea accessions primarily originating from Kazakhstan. Almost all (92%) chickpea germplasms from 13 in total in Cluster C had alleles of the TA142 marker with a 155 bp size band. Most of the samples in Cluster C were of Syrian (ICARDA) origin.

### 3.3. Population Structure Analysis

**Figure 3:**
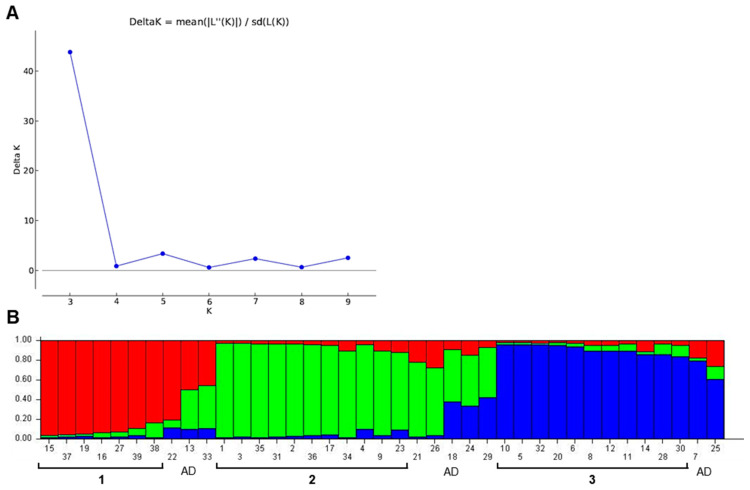
Population structure analysis of 39 chickpea accessions. (**A**) Delta K showing the number of populations. (**B**) Bar plot of populations sorted by kinship matrix. Subgroup 1 is designated as a red zone, subgroup 2 is a green zone, and a blue zone shows subgroup 3; subgroup numbers are indicated under black lines. AD, admixed group. The ID numbers of the 39 studied accessions are indicated in the *x*-axis, and their full names are present in Table 1. The *y*-axis corresponds to the membership coefficient of each genotype.

The population structure of 39 chickpea accessions using the nine SSR markers was estimated using STRUCTURE 2.3.4 Chickpea accessions were split into three subgroups according to the maximum likelihood and delta K (ΔK) values (Figure 3A). Based on a membership coefficient threshold of 0.80, 7 accessions were clustered in subgroup 1, 11 accessions were in subgroup 2, whereas 11 accessions were in subgroup 3, and 10 accessions were retained in the admixed group (AD). Subgroup 1 included most accessions with European origins. Subgroup 2 contained seven accessions from the Soviet Union and two from Syria. In subgroup 3, over half of the accessions (6 out of 11) were released by ICARDA (Syria) and 4 chickpea accessions were from Kazakhstan. This subgroup 3 comprised accessions mostly with an early flowering time (Figure 3B).

Population structure analysis showed clear a distribution of chickpea accessions among the three large subgroups, similarly to the results of cluster analysis. At the same time, chickpea genotypes were identified as admixture accessions during the population structure analysis that contained the genomic content of all three subgroups. These ten admixed chickpea accessions are as follows in the order of occurrence in Figure 3B: 22, F03-34/1; 13, 30236; 33, Kamila; 21, F02-70; 26, F97-24; 18, 34-B; 24, F-92-52; 29, F-97-60; 7, 30121; and 25, F97-147.

### 3.4. Correlation Analysis

**Figure 4:**
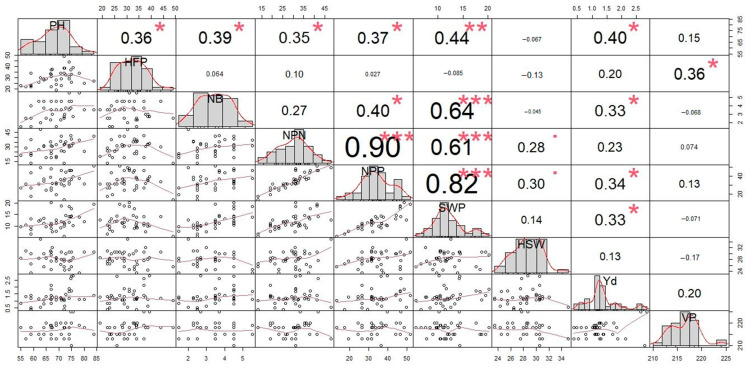
Correlation coefficients among the nine studied traits in 39 chickpea accessions in the germplasm collection (autumn sowing). Traits: PH, plant height (cm); HFP, height to first pod (cm); NB, number of branches; NPN, number of productive nodes; NPP, number of pods per plant; SWP, seed weight per plant (g); HSW, 100 seed weight (g); Yd, yield (t/ha); VP, vegetation period (days). Units of measured traits are shown on the perimeter of the figure. For each trait, the histogram distribution is present in the diagonal squares. Below the diagonal, scatterplots for correlations between trait pairs are shown as dots with corresponding curve lines for correlations. Above the diagonal, values of the correlation between trait pairs are presented. Higher correlation values are indicated by the bigger font and accompanied by red asterisks showing the level of significance of the correlations, which is as follows: * 0.01 < *p* < 0.05; ** 0.001 < *p* < 0.01; and *** *p* < 0.001.

**Figure 5:**
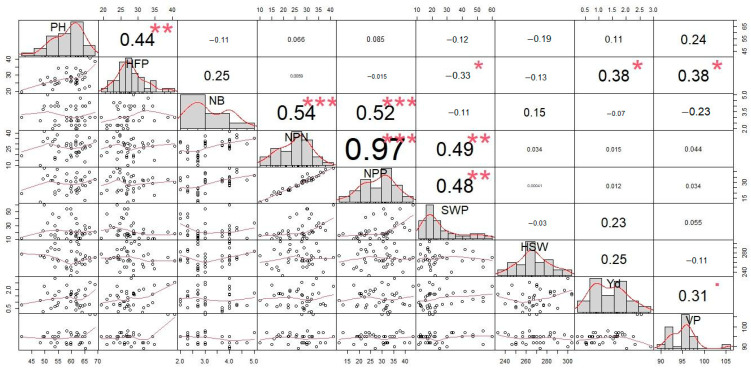
Correlation coefficients among the nine studied traits in 39 chickpea accessions in the germplasm collection (spring sowing). Traits: PH, plant height (cm); HFP, height to first pod (cm); NB, number of branches; NPN, number of productive nodes; NPP, number of pods per plant; SWP, seed weight per plant (g); HSW, 100 seed weight (g); Yd, yield (t/ha); VP, vegetation period (days). Units of measured traits are shown on the perimeter of the figure. For each trait, the histogram distribution is presented in the diagonal squares. Below the diagonal, scatterplots for correlations between trait pairs are shown as dots with corresponding curve lines for correlations. Above the diagonal, values of the correlation between trait pairs are presented. Higher correlation values are indicated by the bigger font and accompanied by red asterisks showing the level significance of the correlations, which is as follows: * 0.01 < *p* < 0.05; ** 0.001 < *p* < 0.01; and *** *p* < 0.001.

During autumn sowing, the correlation analysis of studied yield-related traits showed significant accessions (Figure 4). PH was significantly correlated with the HFP (r = 0.36), NB (r = 0.39), NPN (r = 0.35), NPP (r = 0.37), SWP (r = 0.44), and Yd (r = 0.40). During spring sowing, the relationship between PH and HFP had a stronger correlation (r = 0.44) (Figure 5). In contrast, other traits associated with PH in spring sowing showed non-significant correlations compared to those in autumn sowing.

During autumn sowing, the SWP trait was associated with yield components mostly via NPP (r = 0.82), NB (r = 0.64) and NPN (r = 0.61). The NPP trait was highly correlated with NPN (r = 0.90). In contrast, in spring sowing, the same SWP trait showed a lesser association with NPP (r = 0.48), NPN (r = 0, 49) and NB (r = 0.52) but that between SWP and NPP was stronger (r = 0.97) in spring sowing compared to autumn sowing. The traits NB and NPN were also positively interrelated (r = 0.54). Neither the length of VP nor HSW affected any yield components in this study.

A correlation analysis of yield components in the chickpea accession after autumn sowing showed that SWP was positively related to yield, but it was significantly dependent on NPP (r = 0.82), which in turn is determined by NPN (r = 0.90). The SWP in the autumn sowing experiment was related to NB, NPN, and PH. These traits showed maximal values in tall and branched plants. Such chickpea plants can be adapted to over-winter growth, using early spring precipitation and avoiding drought stress during reproductive organ development, which is sensitive to heat and lack of moisture.

### 3.5. Marker–Trait Association (MTA) Analysis between SSR Markers and Morphological Traits

The association analysis between SSR markers and the morphological yield-related traits in the studied chickpea germplasm revealed a number of informative associations between markers and the following important traits: HFP, NPN, NPP, HSW, SWP, PH, and NB (Table 5).

As a result of marker–trait association analysis, 23 MTAs were revealed in experiments across two years in two sowing seasons (four environments). Among them, six markers, TA14, TA46, TA71, TA142, NCPGR4, and NCPGR7, were identified with an association in total, in eight out of the nine studied traits. Marker NCPGR7 was associated with four traits (SWP, HSW, NPP, and Yd) while other markers were associated with three traits.

The association of most markers identified in the current study was consistent across sowing years except for TA14 and NCPGR4. TA142 associated with traits NPN and NPP was consistent in autumn across two years (2016 and 2017). Alleles of the TA142 marker with an amplicon size for 155 bp were observed in most accessions in Cluster C as well as in subgroup 3, and these chickpea genotypes had high NPN. In autumn sowing, it reached more than 40 productive nodes in some accessions (30113, 30226, 28-B, and F97-63) (Supplementary Material S3). Marker TA71 was associated with the traits HSW and SWP in the autumn and spring of two years (2016 and 2017, respectively). Marker NCPGR7 was linked with four traits; three of them (NPP, SWP, and Yd) were observed in one environment (autumn, 2016), while the association with HSW was observed in two environments (spring, 2016 and 2017). The alleles of the NCPGR7 marker with an amplicon of 211 bp were associated with Yd, and it was mostly observed in accessions of Cluster B and subgroup 2. The average yield value in chickpea accessions in this Cluster B was approximately 1.50 t/ha, which exceeds the average yield value of many chickpea accessions in other clusters. Marker TA46 was linked with several traits such as NPP, PH, and NPN, and it was consistently identified in the spring sowing experiments during both years of study. The allele of the NCPGR4 marker with an amplicon size of 194 bp, associated with Yd, was observed in five out of seven accessions in subgroup 1. These five accessions were also identified in Cluster A, which contains 12 accessions. However, population structure analysis identified the other seven accessions in Cluster B as Ads. Accessions with this NCPGR4 allele (194 bp) showed a high Yd across both spring and autumn sowing seasons, with an average of 1.73 and 1.85 t/ha, respectively.

### 3.6. Potential Candidate Gene Identification

**Figure 6:**
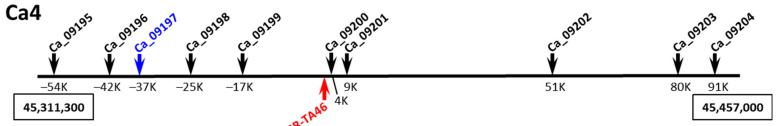
The genetic fragment on chromosome Ca4; a physical map of the cv. Frontier, with the localized position of SSR marker TA46 indicated in red. Five closest genes from both sides from TA46 are indicated by arrows with the IDs above. Distances from TA46 in 1000 bp (K) are shown under the line. Numbers in boxes represent the position of the genetic region on chromosome Ca4. The gene *Ca_09197* indicated in blue and encoding early flowering protein is the most suitable candidate involved in the reaction of chickpea plants after seed sowing in autumn.

Among all studied SSR markers, three of them, TA14, TA46, and TA142, were identified as mostly promising for an association with better growth and yield-related traits. However, only one SSR marker, TA46, showed a perfect match of both primers in chromosome Ca4, in the genome of the chickpea cv. Frontiers (Supplementary Materials S4). An analysis of the genetic fragment near to the position of the identified TA46 marker revealed 10 surrounding genes, with 5 each in the distal and proximal side from TA46 (Figure 6). These 10 genes were distributed non-equally in both sides, with 5 genes (*Ca_09195-09199*) in the distal part of the genetic fragments covering about 54 K bp and these genes located with a relatively similar interval. In contrast, in the proximal side from TA46, five other genes (*Ca_09200-09204*) had some clustering and big gaps between genes covering a fragment of about 91K on the chromosome. Based on the annotations presented on the chickpea genome of cv. Frontiers, only one gene, *Ca_09197*, indicated in blue (Figure 6), can be estimated as the most promising candidate for the reaction of chickpea plants for autumn seed sowing. It encodes early flowering protein, which is involved in the control of plant growth and transition from the vegetative to reproductive stage.

## 4. Discussion

The genetic diversity in chickpea germplasm collections is important for developing a breeding strategy in specific agro-ecological conditions. The great resolving power of SSR markers for assessing the genetic variability and relationships among the chickpea accessions was found earlier [54]. In the frame of international breeding programs, researchers from International Crops Research Institute for the Semi-Arid Tropics (ICRISAT) in India and from ICARDA in Syria assessed the genetic diversity of 3000 chickpea accessions using 35 SSR markers [36]. The markers were selected by the authors based on the criteria of score quality, genome coverage, quality of amplified products, and polymorphism information content. The presented set of SSR markers has a wide range of applications, especially for studying genetic diversity in chickpea.

The assessment of the genetic diversity of the studied germplasm collection included some of the markers used by many other authors [31,32,33,34,35,36]. The polymorphism of SSR markers used for the current analysis of the chickpea germplasm was lower in most cases due to the difference in the number of compared genotypes. At the same time, in this chickpea germplasm collection in Kazakhstan, unique alleles in several SSR markers were identified that were not previously described. Thus, many chickpea genotypes in this germplasm collection in Kazakhstan had specific alleles of the following SSR markers: TA14, TA46, TA71, TA76s, and TA142 (Table 3).

Polymorphism information content, PIC, is one of the most important indexes in estimating primer efficiency [55]. The value of PIC reflected the allele diversity among the studied genotypes. The high polymorphism in microsatellites showed wide diversity, suggesting that many mutations occurred in microsatellites’ genetic regions [56].

UPGMA is a type of hierarchical clustering method that is more reliable and most commonly used [57]. However, UPGMA would not be reliable if only a small number of data were used. Clearly, UPGMA results are dependent on the sample size and number of markers. However, UPGMA can still be robust with few markers if selected according to their high information content [58]. Additionally, a Bayesian approach, which is more robust than clustering analysis, is implemented to infer more details of germplasm origin [59,60].

Clustering via the UPGMA divided all accessions into three clusters. There was no clear differentiation of samples according to geographic origin; for instance, Cluster A contained samples from different geographic origins, such as Kazakhstan and European countries. Cluster B mostly had accessions from the former Soviet Union, including Azerbaijan, Russia, Moldova, Ukraine, and Kazakhstan, while two others originated from Syria. Cluster C comprised six samples from ICARDA and five samples from Kazakhstan. Similar results were obtained in many studies including chickpea [61,62]. The current results confirm that clustering using molecular markers does not always divide samples by their origins, and it depends on various factors such as pedigree and breeding, as well as the exchange of genetic materials with gene flow and mutations [63,64].

The results from population structure analysis also separated chickpea samples into three subgroups, like under UPGMA methods, giving relatively similar discrimination in the results obtained from UPGMA clustering. Interestingly, subgroup 3 had almost the same accessions as Cluster C did in UPGMA methods. Most of these accessions showed an early flowering time associated with alleles of marker TA142 with an amplicon size of 155 bp, and this was found among accessions in Cluster B and subgroup 2, which are associated with the HFP trait in the current study. However, another allele of the TA142 marker with a 147 bp amplicon was reported as being associated with the PH trait [65].

Significant and positive correlations of seed yield with PH and NPP have also been established earlier [66]. The number of secondary branches per plant and PH positively correlated with SWP [67]. The seed yield was positively and statistically significantly correlated with biological yield, the harvest index, PH, NB, and NPP, while it was negatively and statistically significantly related to HSW [68]. The authors concluded that HSW should be used in breeding for larger seeds. A significant positive correlation was found between the NPP with PH and NB in chickpea plants sown in autumn in cold regions of Iran [69].

The obtained correlation results confirm that chickpea seed yield is highly dependent on PH, branching, and the setting of a large NPP. Thus, chickpea PH in experiments with seeds sown in autumn varied from 55 to 84 cm, while chickpea plants sown in spring were significantly shorter, from 41 to 67 cm. NB in plants from seeds sown in autumn varied widely between 1.5 and 5.5 branches, while in spring sowing experiments, plants showed slightly less variability in intervals of two to five branches. A similar tendency was observed with the NPP trait, which varied from 13.2–41.8 to 10.0–41.2 in autumn and spring sowing experiments, respectively. For the NPP trait, the range was wider, at 13.5–51.5 in autumn-sown seeds compared to that of those in the spring sowing season (11.3–43.3). Plants of accessions in Cluster C and subgroup 3 had taller PH values, and higher values for both NPP and NB traits.

Global climate warming leads to ‘aridization’, which is characterized by a lack of moisture, increased temperature, and unfavorable plant growth and development factors. Thus, according to [70], the average warming trend in the winter season across Kazakhstan is 0.26 °C every 10 years. The winter temperature trend was positive in all regions, but the most noticeable instance of warming, by 0.41–0.55 °C per 10 years, was noted in southern Kazakhstan, including the Turkestan region.

Winter seeding is recommended for high yields due to chickpea plants becoming taller, branching well, having larger seeds, and producing larger seed yields [14,71]. The time-shift for sowing chickpea from spring to autumn has an advantage in the regions of Central Asia, including the south and southeast of Kazakhstan, which are characterized by hot summers with low rainfall. Chickpea germplasms must be identified and selected with suitable adaptation for autumn sowing and growing over winter in such regions, suggesting an urgent task. Therefore, there is a strong demand to study germplasm collections, select adapted genotypes, and include them in the hybridization process with the subsequent genotyping of promising chickpea breeding lines using traditional breeding and MAS methods.

**Figure 7:**
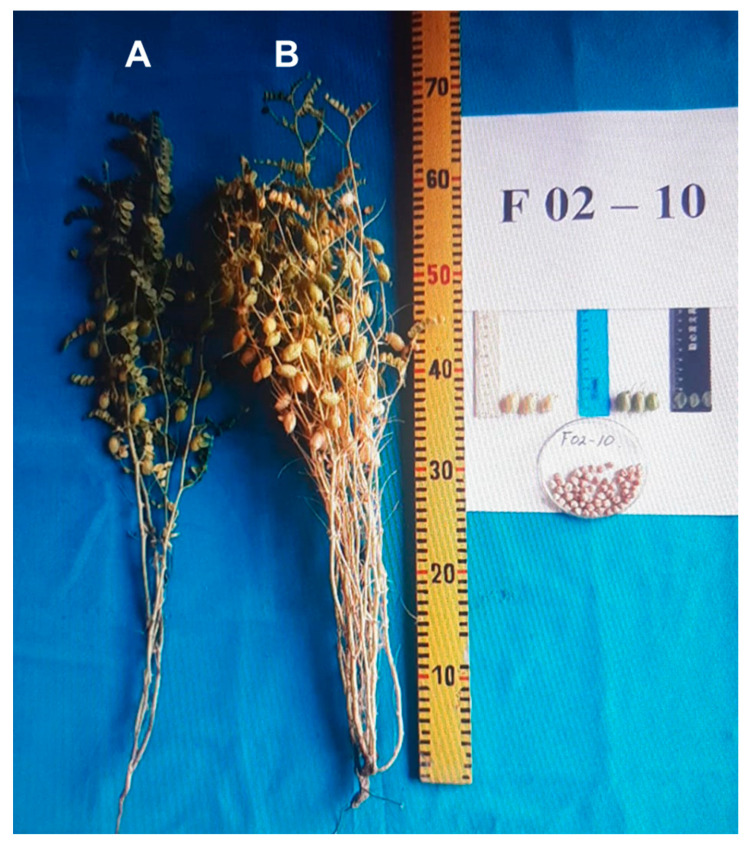
Example of individual plants of chickpea accession F02-10 collected from research fields. Plants were grown from spring-sown seeds in 2017 (**A**) and from autumn-sown seeds in 2016 (**B**). Experiments were carried out in Almaty district, Kazakhstan.

The study of chickpea germplasm collections for adaptability to autumn sowing has not yet been carried out in Kazakhstan. Similarly, the cultivation of cold-tolerant chickpea genotypes released in Uzbekistan shows the advantage of autumn-sown chickpea. These plants are taller, grow better, and are suitable for combine harvesting, producing higher seed yields and bigger biomass for animal feed [16]. A high yield and its stability for the autumn sowing of chickpea accessions were associated with the efficient use of precipitation, avoiding drought stress compared to that experienced by plants grown in spring sowing seasons [69,72]. This point can be illustrated by one of many of our results obtained in this study, where chickpea accession F02-10 showed early-matured, bigger, and more productive plants from autumn-sown seeds compared to those from spring-sown seeds (Figure 7).

In the current study, six markers were found to be associated with eight yield-related traits according to the MLM (K+Q) results. These associations were mainly observed in the new alleles identified in the present study. For example, two new alleles of TA142 with amplicon sizes of 147 and 155 bp, revealed in this study, were associated with HFP (147 bp), and NPN and NPP traits (155 bp). Other alleles of the same SSR marker, TA142, were also associated with PH, early vigor growth, and Ascochyta blight resistance [32,65].

New alleles of the TA46 marker with amplicons of 155 and 162 bp were found in this study, and they were associated with the traits NPP, NPN, and PH. The MTA for TA46-NPP was confirmed in our study, and it was exactly the same as that reported previously by other authors [32,73]. Additionally, the SSR marker TA46 was very effective and associated with many other important agronomic traits such as HSW, NPP, relative yield, seed number per plant, harvest index, and Fusarium wilt incidence [32,73]. SSR marker TA71 with two alleles with amplicons of 230 and 237 bp, identified as new alleles in this study, were associated with the traits HFP, SWP, and HSW. The NCPGR4 and NCPGR7 markers revealed one MTA associated with seed yield. Although only one MTA for NB was observed for TA14 in the current study, this marker was associated with several traits such as PH, SWP, HSW, seed diameter, and Ascochyta blight resistance [32,65,73,74,75,76].

The identified candidate gene, *Ca_09197*, encoding early flowering protein, and closely linked to SSR marker TA46 in chromosome Ca4, has to be carefully evaluated as the most promising gene for the reaction of chickpea plants for autumn seed sowing. Early flowering is a very important trait for chickpea plants and a deeper analysis of the *Ca_09197* gene can be very promising for suitable molecular marker development and MAS application in chickpea breeding for autumn seed sowing seasons.

## 5. Conclusions

The genetic germplasm collection used in chickpea breeding in South-Eastern Kazakhstan showed high allelic diversity based on the analysis of molecular markers. The average genetic diversity for all polymorphic SSR loci was 0.69. The markers TA14, TA22, TA46, TA71, and NCPGR4 were the most effective loci among the 11 used in this study. The markers used clearly differentiate the germplasm collection and are suitable for the genotyping of each chickpea accession. This is important for preserving the gene pool and for the future molecular analysis of prospective breeding lines. The correlation analysis of relationships between important components of yield revealed the associations of chickpea seed yield with PH, branching, and the setting of a large NPP. These results were most evident during the autumn sowing of chickpeas in the southern and south-eastern regions of Kazakhstan. Results of marker–trait association analysis showed that six SSR markers, TA14, TA46, TA71, TA142, NCPGR4, and NCPGR7, were associated with eight out of the nine traits studied in this work. The association of markers was consistent across sowing years except for markers TA14 and NCPGR4. The identified relationships between alleles of SSR markers and yield components show great importance for the selection of prospective genotypes. This knowledge can accelerate the production process for new high-yielding chickpea varieties based on molecular markers and MAS, including one of the promising candidate genes, *Ca_09197*, in chromosome Ca4, encoding early flowering protein.

## Figures and Tables

**Table 1:** Origin and morphological characteristics of studied chickpea germplasm collection.

Entry N	Name of Accessions	Origin	Morphological Characteristics				
			Time to Flowering	Flower Color	Seed Color	Seed Shape	Seed Ribbing
1	882	Türkiye	medium	white	yellow	round	absent
2	1221	Russia	medium	white	grey-brown	round to angular	weak to medium
3	1229	Moldova	medium	white	grey-brown	round to angular	weak to medium
4	12124	Kazakhstan	medium	white	grey-brown	round to angular	weak
5	30112	ICARDA	early	white	grey-brown	round to angular	weak
6	30113	ICARDA	early	white	grey-brown	round to angular	medium
7	30121	ICARDA	early	white	grey-brown	round to angular	medium
8	30128	ICARDA	early	white	grey-brown	round to angular	medium
9	30130	ICARDA	early	white	grey-brown	round to angular	medium
10	30201	ICARDA	early	white	grey-brown	round to angular	medium
11	30226	ICARDA	early	white	grey-brown	round to angular	medium
12	30232	ICARDA	early	white	grey-brown	round to angular	medium
13	30236	ICARDA	early	white	grey-brown	round to angular	medium
14	13-B	Syria	early	white	rose-brown	round to angular	weak to medium
15	28-B	Ukraine		white	brown	angular	strong
16	31-B	Türkiye	medium	white	grey-brown	round to angular	medium
17	33-B	Syria	medium	white	grey-brown	round to angular	weak to medium
18	34-B	Morocco	medium	white	grey-brown	round to angular	medium
19	Ezbsen Sponishe	Germany	medium	white	grey-brown	round to angular	weak to medium
20	F02-10	Kazakhstan	early	white	grey-brown	round to angular	medium
21	F02-70	Kazakhstan	early	white	grey-brown	round to angular	medium
22	F03-34/1	Kazakhstan	medium	white	grey-brown	round to angular	weak to medium
23	F103	Kazakhstan	medium	white	rose-brown	round to angular	weak to medium
24	F92-52	Kazakhstan	medium	white	rose-brown	round to angular	medium
25	F97-147	Kazakhstan	medium	white	rose-brown	round to angular	medium
26	F97-24	Kazakhstan	medium	white	grey-brown	round to angular	weak to medium
27	F97-25-01	Kazakhstan	medium	white	rose-brown	round to angular	weak to medium
28	F97-52	Kazakhstan	medium	white	grey-brown	round to angular	weak to medium
29	F97-60	Kazakhstan	medium	white	grey-brown	round to angular	weak to medium
30	F97-63	Kazakhstan	medium	white	grey-brown	round to angular	medium
31	F98-130	Kazakhstan	early	white	grey-brown	round to angular	strong
32	F99-73	Kazakhstan	early	white	rose-brown	round to angular	medium
33	Kamila	Kazakhstan	medium	white	yellow	round to angular	weak
34	Liniya-7B	Russia	medium	white	grey-brown	round	absent
35	Liniya-8B	Russia	medium	white	grey-brown	round	absent
36	Luch	Kazakhstan	medium	white	grey-brown	round	absent
37	Malhotra	Syria	late	purple-pink	brown	angular	strong
38	S-35	Russia	medium	white	grey-brown	round to angular	medium
39	Vysokoroslyj	Azerbaijan	medium	white	grey-brown	round to angular	weak to medium

**Table 2:** Microsatellite markers employed for genotyping the chickpea germplasm collection [32,33].

SSR Marker	Motif	Primer Sequence	LG	Amplicon (bp)
TA14	(TAA)22 n(TAA)4 T(A)3 n(AAT)5 n(A)3 n(GAT)4 (TAA)5	F: TGACTTGCTATTTAGGGAACA R: TGGCTAAAGACAATTAAAGTT	6	144, 263–278
TA22	(ATT)40	F: TCTCCAACCCTTTAGATTGA R: TCGTGTTTACTGAATGTGGA	4	203–278
TA46	(TAA)22	F: TTTATTGCAATAAAACTCATTTCTTATC R:TTCTTTTTGTGTGAAAAAAAAATATAGTGA	6	69, 127–154
TA71	(AAT)32	F: CGATTTAACACAAAACACAAA R: CCTATCCATTGTCATCTCGT	5	138, 184–223
TA76s	(AAT)7(AAT)4 [ACT(AAT)11]2 n(AAT)3 n(AAT)2 (ATT)5	F: TCCTCTTCTTCGATATCATCA R: CCATTCTATCTTTGGTGCTT	3	165, 203, 206, 212, 218
TA142	(TTA)15	F: TGTTAACATTCCCTAATATCAATAACTT R: TTCCACAATGTTGTATGTTTTGTAAG	7	84, 125–140
NCPGR 4	(CT)16	F: TTACAGCTTGTGCTCAG R: AGTCAGATTCTTATCCGA	6	180, 194, 196
NCPGR 6	(CA)12	F: GACCAAGATTAGTAGAACCT R: TATGTCTACACCTATGCATC	4	249, 251, 255
NCPGR 7	(CA)14	F: GACCAAGATTAGTAGAACCT R: CTTGATAAGGATGAGTCATG	4	217, 219, 223
NCPGR 12	(CT)35	F: CCTTGTTAGTGTGTATAGGT R: GTAATGACCAAGTGAACA	7	213–261
NCPGR 19	(GA)19	F: TCCATTGTAGCTTAGCTTAG R: TCTTACTCTTAGCTTACCTCTT	7	298–312

**Table 3:** Genetic polymorphism and amplicon size of SSR markers in the studied chickpea germplasm collection in KRIAPG, Kazakhstan.

SSR Marker	Amplicon (bp) in the Current Research	Amplicon (bp) in s	Reference
TA14	263, 278, 288, 300, 307	250	[32]
		266, 272, 278	[34]
		266, 272	[31]
		263, 278	[33]
		263, 266, 272, 278	[36]
TA22	195, 203, 209, 218, 227, 236, 239, 245, 251, 263, 278	228	[32]
		203, 209, 212	[34]
		209, 278	[31]
		206, 269	[33]
		203, 206, 209, 212, 269, 278	[36]
TA46	152, 155, 162, 164, 166, 171, 176, 186	152	[32]
		142, 145	[34]
		145, 148, 151	[31]
		127, 154	[33]
		127, 142, 145, 148, 151, 154	[36]
TA71	184, 196, 202, 214, 223, 230, 237	225	[32]
		196, 205, 214	[34]
		196, 202, 223	[31]
		184, 187, 202	[33]
		184, 187, 196, 202, 205, 214, 223	[36]
TA76s	214, 218, 227, 230	206	[32]
		203, 212, 218	[34]
		212, 218	[31]
		212, 218	[33]
		203, 212, 218	[36]
TA142	143, 147, 155, 174	135	[32]
		131	[33]
		125, 128, 137	[31]
		134, 140	[33]
		125, 128, 131, 134, 137, 140	[36]
NCPGR4	174, 180, 186, 194, 198, 200	180, 194	[34]
		194, 196	[31]
		194, 195, 198	[33]
		180, 194, 196	[36]
NCPGR6	245	249, 251, 255	[34]
		251, 255	[31]
		245, 249, 251	[33]
		249, 251, 255	[36]
NCPGR7	211, 214, 217, 221	217, 219, 223	[34]
		219, 223	[31]
		217, 219, 222	[33]
		217, 219,2 23	[36]
NCPGR12	200	225, 259, 261	[34]
		213, 253	[31]
		235, 251, 255	[33]
		213, 225, 253, 255, 259, 261	[36]
NCPGR19	310, 312	298, 300, 308	[34]
		298, 300, 308	[31]
		306, 308, 312	[33]
		298, 300, 308, 312	[36]

**Table 4:** Major allele frequency, number of alleles, gene diversity, and polymorphism in 39 chickpea genotypes.

SSR Marker	Major Allele Frequency	Observed Number of Alleles	Nei’s Gene Diversity (h)	Polymorphism Information Content, PIC
TA14	0.30	5	0.77	0.73
TA22	0.18	11	0.87	0.86
TA46	0.32	8	0.79	0.76
TA71	0.24	7	0.83	0.80
TA76s	0.70	4	0.46	0.42
TA142	0.48	4	0.63	0.57
NCPGR4	0.36	6	0.76	0.72
NCPGR7	0.52	4	0.64	0.58
NCPGR19	0.62	2	0.47	0.36
**Mean**	0.41	5.7	0.69	0.65

**Table 5:** Marker–trait associations with MLM models using TASSEL.

Traits	SSR Marker	Allele (Amplicon, bp)	Environments	*p* Value
PH	TA46	162	Spring, 2016	0.042 *
	TA46	162	Spring, 2017	0.041 *
HFP	TA142	147	Autumn, 2016	0.0086 **
	TA71	223	Autumn, 2017	0.038 *
NB	TA14	278	Autumn, 2017	0.029 *
NPN	TA142	155	Autumn, 2016	0.01 *
	TA142	155	Autumn, 2017	0.0094 **
	TA46	162	Spring, 2016	0.04 *
	TA46	162	Spring, 2017	0.04 *
NPP	TA142	155	Autumn, 2016	0.037 *
	TA142	155	Autumn, 2017	0.025 *
	TA46	162	Spring, 2016	0.02 *
	TA46	162	Spring, 2017	0.02 *
	NCPGR7	211	Autumn, 2016	0.026 *
SWP	TA71	230	Spring, 2016	0.05
	TA71	230	Spring, 2017	0.04 *
	NCPGR7	211	Autumn, 2016	0.046 *
HSW	TA71	237	Autumn, 2016	0.0098 **
	TA71	237	Autumn, 2017	0.01 *
	NCPGR7	221	Spring, 2016	0.03 *
	NCPGR7	221	Spring, 2017	0.04 *
Yd	NCPGR7	211	Autumn, 2016	0.034 *
	NCPGR4	194	Spring, 2016	0.033 *

Note: PH, plant height (cm); HFP, height to first pod (cm); NB, number of branches; NPN, number of productive nodes; NPP, number of pods per plant; SWP, seed weight per plant (g); HSW, 100 seed weight (g); Yd, yield (t/ha). The significance level of the associations is indicated as follows: * 0.01< *p* <0.05; and ** 0.001 < *p* < 0.01.

## Data Availability

The data presented in the manuscript are available in the Supplementary Materials.

## Supplementary Materials

The following supporting information can be downloaded at https://www.mdpi.com/article/10.3390/biom13121722/s1. Supplementary Material S1: PCR products obtained with SSR markers identified during the screening of chickpea collections. Supplementary Material S2: Agronomic traits of chickpea collection samples under spring sowing (average for 2016 and 2017). Supplementary Material S3: Agronomic traits of chickpea collection samples under autumn sowing (average for 2016 and 2017). Supplementary Material S4: Sequences of the fragment of chromosome Ca4 with the identified position of primers for SSR TA46 and a description of 10 genes surrounding SSR marker TA46 in the order occurring on chromosome Ca4 (cv. Frontier sequence).
