# Supplementary material for: SSR Genotyping and Marker–Trait Association with Yield Components in a Kazakh Germplasm Collection of Chickpea (Cicer arietinum L.)
Source: Biomolecules. 2023 Nov 29;13(12):1722. doi: 10.3390/biom13121722 (PMC10741797; doi:10.3390/biom13121722)
Supplement: Supplementary file 1 [file biomolecules-13-01722-s001.zip › biomolecules-2735386-supplementary.pdf]

Supplementary Material

Supplementary Material S1. PCR products obtained with SSR markers identified during the screening of chickpea collections.

N	Names of accessions	TA14	TA22	TA71	TA76s	TA46	TA142	NCPGR4	NCPGR7	NCPGR19
1	882	300/300	226/226	230/230	218/218	164/164	147/147	186/186	211/211	310/310
2	1221	300/300	226/226	223/223	218/218	171/171	147/147	200/200	214/214	312/312
3	1229	307/307	226/226	214/214	218/218	171/171	147/147	186/186	211/211	310/310
4	12124	288/288	195/195	196/196	230/230	164/164	147/147	186/186	211/211	312/312
5	30112	263/263	210/210	202/202	230/230	176/176	155/155	174/174	217/217	312/312
6	30113	300/300	210/210	202/202	230/230	171/171	155/155	174/174	221/221	312/312
7	30121	263/263	246/246	214/214	230/230	176/176	155/155	174/174	214/214	310/310
8	30128	307/307	203/203	184/184	230/230	176/176	155/155	186/186	221/221	310/310
9	30130	307/307	236/236	196/196	227/227	176/176	147/147	200/200	211/211	310/310
10	30201	263/263	203/203	202/202	230/230	171/171	155/155	186/186	217/217	312/312
11	30226	263/263	195/195	184/184	230/230	164/164	155/155	186/186	221/221	312/312
12	30232	288/288	217/217	214/214	230/230	171/171	155/155	186/186	217/217	310/310
13	30236	263/263	210/210	196/196	230/230	176/176	147/147	186/186	214/214	310/310
14	13-B	278/278	217/217	202/202	230/230	152/152	155/155	186/186	214/214	312/312
15	28-B	278/278	240/240	202/202	230/230	152/152	143/143	194/194	214/214	310/310
16	31-B	278/278	240/240	214/214	230/230	164/164	147/147	194/194	214/214	310/310
17	33-B	278/278	226/226	196/196	230/230	162/162	147/147	186/186	211/211	312/312
18	34-B	263/263	251/251	223/223	230/230	164/164	155/155	200/200	211/211	312/312
19	Ezbsen Sponishe	263/263	210/210	214/214	230/230	176/176	143/143	194/194	214/214	310/310
20	F02-10	278/278	217/217	202/202	227/227	171/171	155/155	174/174	217/217	312/312
21	F02-70	278/278	195/195	237/237	230/230	171/171	147/147	194/194	211/211	310/310

22	F03-34/1	263/263	240/240	237/237	230/230	166/166	143/143	180/180	214/214	312/312
23	F103	263/263	226/226	214/214	230/230	171/171	147/147	174/174	211/211	310/310
24	F92-52	278/278	217/217	237/237	230/230	171/171	147/147	186/186	214/214	312/312
25	F97-147	307/307	217/217	230/230	230/230	176/176	155/155	200/200	214/214	310/310
26	F97-24	300/300	195/195	230/230	230/230	171/171	147/147	200/200	214/214	310/310
27	F97-25-01	278/278	240/240	214/214	230/230	176/176	147/147	198/198	214/214	310/310
28	F97-52	288/288	246/246	214/214	218/218	176/176	155/155	186/186	221/221	310/310
29	F-97-60	307/307	210/210	237/237	218/218	171/171	155/155	186/186	214/214	310/310
30	F97-63	278/278	246/246	230/230	218/218	176/176	155/155	186/186	214/214	312/312
31	F98-130	263/263	210/210	237/237	214/214	185/185	147/147	186/186	211/211	310/310
32	F99-73	263/263	246/246	202/202	230/230	166/166	155/155	174/174	217/217	312/312
33	Kamila	263/263	203/203	223/223	230/230	166/166	143/143	200/200	211/211	310/310
34	Liniya-7B	278/278	240/240	237/237	214/214	158/158	147/147	186/186	211/211	310/310
35	Liniya-8B	300/300	226/226	223/223	218/218	185/185	147/147	186/186	214/214	310/310
36	Luch	263/263	264/264	223/223	218/218	171/171	147/147	194/194	211/211	312/312
37	Malhotra	278/278	277/277	202/202	230/230	164/164	143/143	194/194	214/214	310/310
38	S-35	300/300	226/226	196/196	230/230	152/152	143/143	180/180	214/214	310/310
39	Vysokoroslyj	263/263	236/236	230/230	230/230	171/171	143/143	194/194	214/214	310/310

Supplementary Material S2. Agronomic traits of chickpea collection samples under spring sowing (average for 2016 and 2017)

N	Accession names	PH	HFP	NB	NPN	NPP	SWP	HSW	Yd	VP
1	882	53.7	26.7	2.7	17.3	18.7	13	25	0.66	95
2	1221	58.3	34.7	2.7	15	17.7	11	24.67	2.33	91
3	1229	61.7	24.3	2.3	22.3	22.3	15.5	26.67	1.41	95
4	12124	63	21	2.3	22	22.7	22	26	0.56	91
5	30112	54.7	24.7	4.3	38	42	23	26.67	0.74	92
6	30113	66.7	23	4.3	35.7	39.7	27	27	0.83	92
7	30121	64.3	27.3	4	25.3	27.3	16.5	26.6	0.68	95
8	30128	49.3	24.3	3.7	32	33.3	22	27.33	0.23	95
9	30130	58	31.7	3.7	28.3	31.7	19	25	0.55	92
10	30201	64	31.3	3	31	32.3	18	25.67	0.22	92
11	30226	63.3	33	4.7	30	32	12	27.67	0.75	92
12	30232	61.7	34.3	4	32.3	34.3	21	25.67	1.63	95
13	30236	55	34.3	4	28	28.7	11	26.33	0.97	95
14	13-B	61.7	25.7	2	31	34.3	40	26.33	1.06	95
15	28-B	69	38.7	3	32.7	32.7	20.5	27	2.4	106
16	31-B	65	22	2.3	28	35.3	30	26.8	1.56	95
17	33-B	48.3	27	2.7	10	11.3	15.5	27.33	1.14	92
18	34-B	65	28.7	2.7	13	13	18	25.67	0.79	97
19	Ezbsen Sponishe	62	24.7	3	37	39	33	24	0.88	97
20	F02-10	62	26.7	2.7	18.7	20	18	30.33	1.69	95
21	F02-70	59.7	29	4	27.7	30.3	18	30.33	1.58	92
22	F03-34/1	60	26.7	2.3	25.7	26.3	47	28.33	1.86	96
23	F103	62.7	30.3	2.7	22.7	24.7	12	29.67	1.96	96
24	F92-52	61.7	27.7	3.7	28.7	30.7	14	26.33	0.38	95

25	F97-147	62	27	2	22	23.3	18	28.2	0.78	95
26	F97-24	41.3	19.3	3	18.7	19.3	12	27.67	0.76	95
27	F97-25-01	58.3	27	3	30.3	37.7	54	26.33	1.34	98
28	F97-52	52.3	26	4	28.7	31.7	17	29	0.5	92
29	F-97-60	53.7	24	2.3	16	20.7	17	28.67	1.52	94
30	F97-63	60	28.7	2.7	18	20.7	19	24.33	1.68	96
31	F98-130	53.7	28	4	34	35.3	30	30	2.9	90
32	F99-73	65.3	31.7	4	26.3	27	25	27.33	2.01	89
33	Kamila	59.7	28.7	2.7	19.7	24.3	22	23.33	1.62	97
34	Liniya-7B	51	28.7	5	35	36.3	34	28.33	1.73	97
35	Liniya-8B	49.7	24	4	41.3	43.3	60	27	2.25	95
36	Luch	53	21	2	32	31.3	47	26.67	1.24	95
37	Malhotra	67	40.7	3	27.7	30	18	26.33	2.55	106
38	S-35	59.7	26	3	30	30.7	54	25	1.03	91
39	Vysokoroslyj	55.3	25.3	2.3	22.7	22.7	42	28.67	1.84	97

Note: PH-plant height (cm), HFP – height to first pod (cm), NB – number of branches, NPN – number of productive nods, NPP – number of pods per plant, SWP – seed weight per plant (g), HSW – hundred seed weight (g), Yd – Yield (t/ha), VP – vegetation period (days).

Supplementary Material S3. Agronomic traits of chickpea collection samples under autumn sowing (average for 2016 and 2017).

N	Accession names	PH	HFP	NB	NPN	NPP	SWP	HSW	Yd	VP
1.	882	59	23	2.5	21.2	26.5	8.4	26.5	0.47	218
2.	1221	74	31	5.5	31.2	46.5	18.4	24.5	1.7	215
3.	1229	71	23	3.5	26.2	31.5	11.9	28	1.2	215
4.	12124	67	22	3.5	26.2	26.5	10.5	27.5	0.8	213
5.	30112	62	26	4.5	27.2	31.5	12.2	28.5	1.14	215
6.	30113	84	27	3.5	41.2	51.5	19.5	30.5	1.38	215
7.	30121	69	24	1.5	19.2	19.5	7.7	27.5	1.25	213
8.	30128	59	22	2.5	13.2	17.5	6.6	26.5	0.38	218
9.	30130	64	29	4.5	31.2	34.5	13.4	25.5	0.79	215
10.	30201	72	34	2.5	25.2	29.5	11.4	27.5	0.38	213
11.	30226	71	29	4.5	41.2	46.5	18.7	30.5	1.08	215
12.	30232	75	33	4.5	38.2	46.5	17.6	25.5	1.74	22
13.	30236	59	34	1.5	23.2	26.5	9.8	23.5	1.17	218
14.	13-B	72	38.7	2.7	32.4	35.3	10	28	1.11	218
15.	28-B	74.7	37	3.7	41.8	46.7	14.2	27.67	2.65	225
16.	31-B	69	19	3.5	20.2	31.5	12.7	28.7	1.26	220
17.	33-B	59	23.7	4	37.8	44	13	30	1.39	215
18.	34-B	72	49	2.5	13.2	13.5	5.6	25.5	1.01	218
19.	Ezbsen Sponishe	72.3	38.7	3	33.1	36.7	11	24.33	0.62	218
20.	F02-10	74	44	4.5	30.2	33.5	14.2	30.5	1.19	218
21.	F02-70	67	37	4.5	19.2	26.5	11.2	30.5	2.14	213
22.	F03-34/1	66.7	33.7	2.3	35.4	39	10.5	31	1.15	219
23.	F103	69	28	4.5	32.2	32.5	11.4	28.5	1.06	219
24.	F92-52	62.7	30.3	2	48	49.7	12.6	35	0.54	213

25.	F97-147	80	25	3.5	25.2	31.5	12	30.5	1.1	218
26.	F97-24	55	23	1.5	21.2	26.5	9.6	28.5	0.8	218
27.	F97-25-01	72.7	32.7	3.7	35.4	44.3	14.5	30	1.47	220
28.	F97-52	57	22	2.5	29.2	31.5	10.4	31	1.3	213
29.	F-97-60	67	37	4.5	19.2	26.5	10.4	30.5	1.39	219
30.	F97-63	78	34	5.5	37.2	37.5	13.9	23.5	1.11	219
31.	F98-130	75	26	4.5	31.2	36.5	14.4	30.5	2.78	210
32.	F99-73	74	29	3.5	31.2	31.5	11.7	27.5	1.87	213
33.	Kamila	70.7	34.3	2.3	36.1	36.3	9.3	26.33	1.23	220
34.	Liniya-7B	65	29	2.5	19.2	24.5	9.8	28.5	1.3	220
35.	Liniya-8B	67	31	4.5	31.2	48.5	20.2	29.5	1.83	218
36.	Luch	64	28.3	3.7	30.4	36.7	11.2	30	1.07	218
37.	Malhotra	75.7	39.3	2.7	33.4	39.7	10.2	28.67	2.86	225
38.	S-35	67	33	3.5	23.2	24.5	9	24.5	1.12	215
39.	Vysokoroslyj	67.3	37.7	3	30.8	32	7.5	32	1.26	218

Note: PH-plant height (cm), HFP – height to first pod (cm), NB – number of branches, NPN – number of productive nods, NPP – number of pods per plant, SWP – seed weight per plant (g), HSW – hundred seed weight (g), Yd – Yield (t/ha), VP – vegetation period (days).

Supplementary Material S4.

Sequences of fragment of chromosome Ca4 with identified position of primers for SSR TA46 (indicated in yellow) together with closest gene Ca_09200 (in reverse order). Start-codon is indicated in green and Stop-codon is in red.

```
>cicar.CDCFrontier.gnm1.Ca4 version 1.0 (fragment) for TA46 and gene Ca_09200.
Ca4:45,360,841-45,369,240 forward strand
CTTAAACTTTTTACTTGAAAAGATTGGTTCGTGCTTGCATTCTCTGTAGCTCTATTAGTGCCATAGATCGACAACAATTAGATTTAGAT
TTAGTAAGATAAATATAATAGAGTGAGTCGTGACTTAAAATTTCGAATATTTTTTGAATTTATTGAATTGCTGCTAAAGTTTATTTTAA
GATAAAGATAACATTGAGAAATCTTTAAAAATAGAAAAAATGATATTTGAAATCAAATTTTGAGTTCGAGAGTCGTTTATGGGTAAAAA
GACATCTGTTAAAATACAATTAGCTATAATTAATTATACAAAATTAATGTTAAGTTATATAAATTTATTTATAATTTTTAAAATATAAA
TATAATTCTTTTTGTGAAAAAAAAATATAGTGATAGAAGTATATTTAATTATTATTATTATTATTATTATTATTATTATTATTATTATT
ATTATTATTATTATTATTATTATATTTGACAAGAATCCGATAAGAAATGAGTTTTATTGCAATAAAATGGTTTGACAAATGTTGTTGGT
CCGATGAAGAAAAAAATGATATTGAATACAAAAATATTTATGATGAGTGACTTCGTTGTTAATTTAAATTTGGTTAATAAAATTTGATA
TCAACAAGTTGTCACAACTGAAGTGCCTTAAACTATTACTGGCAATATGTCGCATCTCTGTTGATCTGATTTTATTAGAAGATAATTAT
TATTTAGAGCTTAAGATCATCAAGTTGGCGTAACTCATATGTCTTAAAATTAAGATTAACAAAATATAACATATCAATCAATCTTTTTA
TCCACATTGATTTTGAAGAGCATGGATTTTTATTTTATTAAAAATAATAATAATAAATCTAATAATGAATTTATATGATATATAACCCA
TTAGTGGTTGATGTTGGTTGTAGTTGACCCAATACTTAATAAGAGAATAAAATATTAACTATTATAACAAACAAAAGAACTAAAAGAAT
TGAATAAAACTTAAACTAAATCAAAAGTTCGAATGTCTACTCGAATGTCTGAAATGAGAGCAAATATATTTTTTAATAGAACAACAAAA
AATGAAAATAAAATAAATGTGATAAATGAAACACAATTTTAGATTGATGTCTTTAGCGATTTTATTGGAGATTTGGGGTGTATAATAAT
TTTTTATTGGTAAAAATTTGTTGCCAAAGACATCACTATAAAAGTTTTGTTCTTATTATTATTTTTTGTTTGATTTATGGTTGTGAGGT
TGAAGAAGACATAAGGAAACACACCAAAATTTCATATTAAAGGTAAAACAAATTCAAATTAAAGGTATAACACTAAAAACAAAACTATG
AAATTATTACATAGACGAGTTTATAAATACTTACCACATAAGCTGGATATTTGTTATACTCATTTGATTTGCTCTTGTTTTGAATTATT
GTATCCTTCCATCACTCATTTCATGTTTCATTTCACTTACTAACAAAAATCTTACTAGATTAAAAATGAAAACAAGCCTTCCTCTGATT
CATTTTTTGCTTTCAGTTTATAAACAAAAATGATAAATCTTTATATCCATGAGATAAAATTGAGTTCTAGATTATTGCTTATTTTTGCA
AAACAAATGAATTTGTTAAGATAAATCTAGAAAAGAGAGGATGCACTTTTTAATTGCTTGCATATATGGTAAAAAAGTTGCAGCTGTTT
TAGGAAGAAAAACAAAATAGGCTAAAAAGGTGCGAAAGAAAAAAATTTATAAGATCACATGTATACAAAAAATAATTGCAGCTATTTTA
GGGGGAAAAGCCAAAGAGGTTGTAAAGATGGTTTGTATAAAAGGAAAAAGTGTAGTTGTAATGATGTAAATCAAGGAGGTTGAAAAGAT
AGAAGAGAATGCTAATGCAAAAAAAAAAAAAAAAACAAAAAACAAAAACCAGAGGCAACCAGAATCAGAGAAAACTAGAACCAAAGATAA
CCAAAGGTAAAACAAGAATAAAACCAAAACCAAATATAATCAGAACTAGAGGCATAATTAGAACCAGAGCCAGAGGCAAAACCAAAGAG
AACCAGAACCAGAACTAGAGGCAAAATTAGAACAAGAGACAAAACTAGAATCAGAGGTAGAACCAAAAAGAACAAGGATCAGAGACAAC
```

TAGAACCAATGATAGAGATAACTAGAGCCAAAACTAGAACCGAAGCCACAATCAGAACCAAAAGGAACCAAAACCAGAGAGCCAAAGAC
ATAACCAGAACTAGAGAAAACTAAAACTATAATCAGAGAAAACCATGAACCTTTTTATAACAACTATTAAAATTAAACGTATGACTTAT
TTTTTTAAAATTTCAATTGACCGAACAAAATTGAAGTACTAGAAAGTGAATATCAACACTTGATCTATTTCATAAAAATGATAATATTA
ACATTTTTTGCCATAAGCAGCCAAAAATCAAATATTTCATGTTTATTTTATTTTTTAACTTCCAAATATTCTCCTGAAATAACCTTTCA
AGTATAACTTTATAAGTGCATACAATTAAGGATGAGTTTTGTTGTCATTGTGAATTTGCTATCATTTTCCAATGCCAAATAGAGTAAGA
AAGGCTAGCTCATTCATTACCTACAAAAAACTACCTTATACTTGCTCTAAAATAATTTTATATATAGATGTTTCAAAAATGATTTTGTA
TTTTAATTTATCAGATTTGACTTTTCATATTTTAAGATAAATTAATAATATTAATAAAAAAATATGTTTGAAAACAAATTAATAAATAT
ATATTATAAATTGAATTAGTTCCTTTTATATATCCGCACCTCTCATTTTCCAACTTTGCATCTCTACATTTTTTTTTCTACCCATTGAA
CTTTTATTTTATGGATTTAATTTTGTGTTTTGCTTTCTCTTATTTTTCACTTTTTCTTTGTTCTCTTATCAACAAGCTTTTTATCTCAC
CGTGTTTTGACATACATTGTATATATATAAAGTAAACACATTCTTTATTCCAAATTTATAAAAAAAATTAAAAAAATACCACCAAATCT
GCAAAAATAAGAAAAATAGCATATAAAAAGTTTTATTTTTTAATTGTTTATTTATTAAATAATTAATAAAAATATCATAAATAAAAAAT
ATAAGAAAAGTACATATTATATATATACACGCCAAAATGGTTATATTTTATTCCAAGTTTAGAAAAAGATGTATTTTATTTATATGCTA
CTACATTTGTCAAAAATAAGATTAACGTTTTATAAGATGCGAAATATGAACATTTTAGAAGTAAATATTTATGAGGAAATTCTATTAAA
CACAATATTTTACACAAAACTTTATTTTACTATTTGTCTATAAATAGTTAATTAAATATGTTCTAATACCTACTAATACACCACAAAGT
TTAATAAATTCATATTTTAAATTTTATTTATTTTGTAATAATTTAATATTTAATGGAGTGGGGATGGGTTAGAGAAAACATGGTTTTTG
TTGTCTTCATTAGAACATAAAATAAACAGCGTTAATAACTCAAACACTATAAATAAAGAATGAACAAATTTGTTTAATGTAGCATGTTG
ATCGTTCCTCAGCATGTCTTCGGTCAAGTTTTTTCCTACCAGCTTATAGATTATTCACAACCACATAAAAAATAAATAAAAGCAAGTCA
ATGACAACTATGGAGTGCACTGCACACAAAGTTGAGTTTGTTACCTGACTTATGAATGAAAATAGCATCTATTATAATTTAAAAGATCA
AAATATTGAACGAAAATAACTAAAGAAATTAAAATAAAACCTTAAAAAAAATGTAAAGAACAAAAGTCTATTTTTAGCCTAGAAATTAA
TATACATCTGCCGTAATAATATTAAGAGGAATCACATGAACTATGAATTTTAGTAATTTATAAAATGATTATTTCAGAGTTCAGAAACG
ATCATTTACTGTGATTTCTAGAAATGCAACACGAGAAGCGTGAGGAGAGAGAAAAGAAGATAAAGAGAATCTTTATGGTTATTGAATGA
GAACAACACAATATTCAACTCCACAAGGTTTGAATTGGAAACACCTCCACGAAACTTTCGTGACCACTCAGCCAGCAAACATGCTTGTG
TCACATTAAACATATATATGTATAAAAATCCTTATTCCCAAACAAAAAGCAAATAACCAAATCTCATGGACCCCACATTATTCATGCCA
TAAACATAAACCTTAAACAAACACTAAAAAAGAAGCATCAAGCTTTGTAATCTTAAATAAATTTTTAAAATTGCTTAATCCAAAAGTTG
GTCTAATAAACTATGTAGTCCTCCTATCTATGGACAAAACACACAATTAACACTAGTAAATAAATTATATTTAAAAACTATAAAATATT
TCACCTAGCTTTCTCCTGTCACACATGTGTCCTTTTACTTAATAATTTCCAATATGTAATTTAATTTTGCTAGAGAATATTGCAACATT
TTGAATTGGTCAATATGGCTTAATGTCAGCATGACTAAAATCAACCCACTCACTAAAGCAAAAACTTGAACCAAAATCACCAACTTTGT
CAGGGCAGTTATTAAGACCATTTTCATGATCATGTGGAAATCCAAATCCAAAATCCAATAACGGGTCCACAGATTCATGATCACCTACT
TGATCTTGAAGCAATTCACAATTGTTGTTCACTTCAATGCTTGAACAATCTTGAGACATTGACATCATAGGATTAATATTACTATTGTT
GTTGTTGTTGTTACTTGTACCAATTTGTGAGACAACAACATCATTGACACTTGGATTATACAATTGATCATGAATTTGGTCAAAACCCA
TGATTTTTTCATTATTGTTAAATCCATAATTAGCATCATAATTATTATTGTCTAGCATAATTGGTAGTGATGAGGGTGGAGAATATTGA

GTTTGTGAAGTAGGAAAATATTGTGGATTTTGAGTTGGTGATGGGGTTGAAGGGAGAAAAGTGTCATTTTGAGTCATTGGTTGGATGGT
AACTTTTCCTGCTATAATTTTCTTCTTCAATTTCGTGTTCCAATAGTTTTTCACATCATTGTCTGTTCTGCCAGGTAGTTTTGATGCTA
TGGCAGACCATCTGCACAAAACACACAAATTTCATCAGTATTCCAACTTTGAGATATATTCCACAAAAGTGCCAAAACTCACTTTTCTA
CAGAGATAAATTTCAATTTAAACAATTATTTTAGGAATTAAAATTATCATTAATTAGAATAATATTAACGACTATTTTATAATTTTGAT
AGAGAGATCTGAATTTTATTTCTTAAAATTATAAGATCAATTTTTTATTGTTGAATTGACATCTCATAAATAAAAATTAAAATAAAACA
AAAATAAAGACAAAAATAAACAATACTATATCTACATAAAAAAAAATTAACCTTAATAATACATGTATATACAAATAATCACGAATATA
ATAAAATAATAATATATTTATAAAAAAAAGAAAAGAATTAAAACTGAATAATACACGTATATATAAATAATCACGACAAGGAGTATGAT
AAAATAACAAATTCTTCCGGCTTACACCCCAATCCAAAATTGAATTTATATGTGTATAACTTGTTTTAAAGTGGTTGAGTAGAACTGAT
TCTAAGTAATTTAGAATTATGATGAATAACTTTTGATTCTAAACATAATTTTGATATTTTTAAATTTATTTTTAAATTAACTTTTATAT
AAATATATTTAAATTATTTTACTTTTAATTTTCTTTTAATCATAATTAAATTTTTGCCAATGCCGTGTGTACCAATTACTAATCATCAA
CTTTAAACAAAAATAATATATGGTACGAATTAAATTAAATATAGTTGTAATAGTTAGCACTTAGCAATCATAAACTTATAACAAAAATT
ATATATTATACAATATTCTCTCAATGAATTACACTTTAATAAAAAAAATCTCAAATTAAATATCATTCACAATTTTTAATATAATTTTA
ATTGTTTTTTTATATACTACTCTTCAATAATTATTATATAAATAATTTTCAAAATATTATTATATTTTATATTAATAATAAAAAAAAC
ACCAATAATTAAAATGAATAATTTATTAAAATATTTTTTAATTATTATATATTTTAATATGTGTATAAAAATCTTAAAAGTAATATTCA
CTGCAACACGGATAAATTAATAAATTAAATAAATTAAATAAATTGGGTATAACAGCTGACTATGATCAATGTTTATAGTGATCCTAATT
TGGAAATTATTATATAGTGATAATATATAGTACTGCTAAATAAAATAATTTGGAAATACTTGCCTGCTACCCATTTGAGCATAGAGGGT
GCAAATGATAGTGTCCTCTTCTTCAGTAAAGCCTCCATGTCTGATGTCTGGCCTAAGATAATTCAACCACCGTAAACGGCAACTCTTGC
CACACCGTTTAAGCCCTACATATCATAAATTAATTATAAATCATTATTCCACCATTCATATATATATATATACTACTAGTTGAAAAAAA
ATGGAATTAGTCTTCAATTGTTTCCCCTCTAACACTTATCCATTGAATTAGTCCATAGAGTCGTAATTTATAGGTGAAAATTTGATCTT
GTAATTAGAAGGGACATATTTATAATTTAACTAATAATATTTTTTTTATTTATAAATTCGACTTAGTCTTCAATTTTTAATTTATAATT
TTTTCTATAATTTAATATTTTTTTTAATAAAGTATGCAAATTTCTTTGACTAAATCAAAATGGTAAACTGTGAGATTCATTAACAGGCA
AGAACAGAGGAACTTCAATCATTGTGTATTATAAAGCAATTTATATCTTTTCCTAGTATAATAAAAAAAAAGCTGGAAATATATGTTTC
TTATAACAGGCTCGGAAATTGCATTTGCAAGAAAATAAAAAAAAAAAATGCAGAAAATACATGTCAATGATAACAACAAATAAACAATCA
TGCTTGCTAGGATAAAAATAAAAATAAAAAATCATTTTCCATCTGAAATTAATTTTTTTCTTTGACTTTGATGTATGTTGAATACCCTT
CAATCGGAAGAAAAATCATGACATTTTGTTTTGACAAAAACAAAATCATGCAATTAAAGATTAAAAAAATTTGCTTAGTGAAAAGGGTA
TCCAAGAAAAGTCATAAAATTTCTCAACAAAAAAAAAAAGTTATAAAAAAATTAAAATAATAATGTATGAAACAATGGGAAGAGCAAAAAA
AAAAAGAAGAAAATACCAGCTTTTTGGGGCAATGCAATCCAACTTCCAGAAGTGCCATGAATCTCTAAATAACTTTTGAGTGTTGCATC
TTCATCAGGAGACCATGGTCCTCTCTTAACATTTTCTTTGTCACAACATGGAGATCTTCCCATGGAATTGAACAGAGAATTGTAGAAAA
ATGACCTTGAAAGCAAGCACCTTTGGTGAAGATGTTGTTATAAAGATGGTGACAATCTAAAGCAGATGAGCCTAGTCAACTACAAGACA
AAACTGTGTGTTATGCATTGTGACAATTAATAAGTCATATATGCATGAAACTGTCAAAAAAGTTTGTGAAATAAATTCAATTTGGTCTA
AAGTTATTGTCTTATTTTATTTTTTTATTGTTGGTCTAAAGTTATAATATTGTTTTATTGTTTATTGTTGGAATACATCTTTATTTAAT

AATTTAACCCATATTTATTGTGACTTTTATTTTTTAATTTTTAAATAATATTTTTATTTGTTTGTGTGATATATATTAATATTATCAAC
TAGCATATTTTTTTTCAACTGATATTATTTTAAGGATACACTCAATAGTTGACTTATCGTACAATTTAAATATAGTAAAATTTTCAGTC
ACTATCCATAGAAATTTATATTTATCATTAACTAAGCTGAATTATATATAATTTATCTGAGACTGTTTACGATTTTGACTATTTTTTAT
TGTTGAAAACTATATATAAATTATTTTTACTTTTTGTAAACTTCCTCTCAATTTTTTTTTACTAAACCTGAATTGGTTGAGTGAAAGGA
ATTTAATTCAGACTTCCTTAAGCATTGTATAGCAGGAAAAACAATTGAAAAATTTAAGTAAAAAGTCTTGAATTCAAAACTCGAAAATT
AACATAACTCTATTAATTTATTAATATTTGTTTATCATTTTTTTTATGGTATGTCACTTTTTTCTCCAAAAATAATTTATGTACATATG
GTATGTAGGATTTTTTTTTTCTAATATGATAATTGCATTCAATGTCTGCATTTATATATTAATATTGTAAAAAGTAAACATTCTTTAAT
TATTGAAAACTTAACTACATGTTATTTTTCATACTTTTTTAATATTAAAATTTATTTCTAGAAGTTGTTTTTAATATTTTTATATTACA

Sequences of fragment of chromosome Ca4 with gene Ca_09200 (in forward order). Start-codon is indicated in green and Stop-codon is in red.

>cicar.CDCFrontier.gnm1.ann1.Ca_09200
cicar.CDCFrontier.gnm1.Ca4:45,365,300-45,368,201 reverse strand
ATGGGAAGATCTCCATGTTGTGACAAAGAAAATGTTAAGAGAGGACCATGGTCTCCTGATGAAGATGCAACACTCAAAAGTTATTTAGA
GATTCATGGCACTTCTGGAAGTTGGATTGCATTGCCCCAAAAAGCTGGTATTTTCTTCTTTTTTTTTTTTGCTCTTCCCATTGTTTCATA
CATTATTATTTTAATTTTTTATAACTTTTTTTTTTGTTGAGAAATTTTATGACTTTTCTTGGATACCCTTTTCACTAAGCAAATTTTTT
TAATCTTTAATTGCATGATTTTGTTTTTGTCAAAACAAAATGTCATGATTTTTCTTCCGATTGAAGGGTATTCAACATACATCAAAGTC
AAAGAAAAAAATTAATTTCAGATGGAAAATGATTTTTTATTTTTATTTTTATCCTAGCAAGCATGATTGTTTATTTGTTGTTATCATTG
ACATGTATTTTCTGCATTTTTTTTTTTTATTTTCTTGCAAATGCAATTTCCGAGCCTGTTATAAGAAACATATATTTCCAGCTTTTTTTT
TATTATACTAGGAAAAGATATAAATTGCTTTATAATACACAATGATTGAAGTTCCTCTGTTCTTGCCTGTTAATGAATCTCACAGTTTA
CCATTTTGATTTAGTCAAAGAAATTTGCATACTTTATTAAAAAAAATATTAAATTATAGAAAAAATTATAAATTAAAAATTGAAGACTA
AGTCGAATTTATAAATAAAAAAAATATTATTAGTTAAATTATAAATATGTCCCTTCTAATTACAAGATCAAATTTTCACCTATAAATTA
CGACTCTATGGACTAATTCAATGGATAAGTGTTAGAGGGGAAACAATTGAAGACTAATTCCATTTTTTTTCAACTAGTAGTATATATAT
ATATATGAATGGTGGAATAATGATTTATAATTAATTTATGATATGTAGGGCTTAAACGGTGTGGCAAGAGTTGCCGTTTACGGTGGTTG
AATTATCTTAGGCCAGACATCAGACATGGAGGCTTTACTGAAGAAGAGGACACTATCATTTGCACCCTCTATGCTCAAATGGGTAGCAG
GCAAGTATTTCCAAATTATTTTATTTAGCAGTACTATATATTATCACTATATAATAATTTCCAAATTAGGATCACTATAAACATTGATC
ATAGTCAGCTGTTATACCCAATTTATTTAATTTATTTAATTTATTAATTTATCCGTGTTGCAGTGAATATTACTTTTAAGATTTTTATA
CACATATTAAAATATATAATAATTAAAAAATATTTTAATAAATTATTCATTTTAATTATTGGTGTTTTTTTTATTATTAATATAAAATA
TAATAATATTTTGAAAATTATTTATATAATAATTATTGAAGAGTAGTATATAAAAAAAACAATTAAAATTATATTAAAAATTGTGAATG

ATATTTAATTTGAGATTTTTTTTATTAAAGTGTAATTCATTGAGAGAATATTGTATAATATATAATTTTTGTTATAAGTTTATGATTGC
TAAGTGCTAACTATTACAACTATATTTAATTTAATTCGTACCATATATTATTTTTGTTTAAAGTTGATGATTAGTAATTGGTACACACG
GCATTGGCAAAAATTTAATTATGATTAAAAGAAAATTAAAAGTAAAATAATTTAAATATATTTATATAAAAGTTAATTTAAAAATAAAT
TTAAAAATATCAAAATTATGTTTAGAATCAAAAGTTATTCATCATAATTCTAAATTACTTAGAATCAGTTCTACTCAACCACTTTAAAA
CAAGTTATACACATATAAATTCAATTTTGGATTGGGGTGTAAGCCGGAAGAATTTGTTATTTTATCATACTCCTTGTCGTGATTATTTA
TATATACGTGTATTATTCAGTTTTAATTCTTTTCTTTTTTTTATAAATATATTATTATTTTATTATATTCGTGATTATTTGTATATACA
TGTATTATTAAGGTTAATTTTTTTTTATGTAGATATAGTATTGTTTATTTTTGTCTTTATTTTTGTTTTATTTTAATTTTTATTTATGA
GATGTCAATTCAACAATAAAAAATTGATCTTATAATTTTAAGAAATAAAATTCAGATCTCTCTATCAAAATTATAAAATAGTCGTTAAT
ATTATTCTAATTAATGATAATTTTAATTCCTAAAATAATTGTTTAAATTGAAATTTATCTCTGTAGAAAAGTGAGTTTTGGCACTTTTG
TGGAATATATCTCAAAGTTGGAATACTGATGAAATTTGTGTGTTTTGTGCAGATGGTCTGCCATAGCATCAAAACTACCTGGCAGAACA
GACAATGATGTGAAAAACTATTGGAACACGAAATTGAAGAAGAAAATTATAGCAGGAAAAGTTACCATCCAACCAATGACTCAAAATGA
CACTTTTCTCCCTTCAACCCCATCACCAACTCAAAATCCACAATATTTTCCTACTTCACAAACTCAATATTCTCCACCCTCATCACTAC
CAATTATGCTAGACAATAATAATTATGATGCTAATTATGGATTTAACAATAATGAAAAAATCATGGGTTTTGACCAAATTCATGATCAA
TTGTATAATCCAAGTGTCAATGATGTTGTTGTCTCACAAATTGGTACAAGTAACAACAACAACAACAATAGTAATATTAATCCTATGAT
GTCAATGTCTCAAGATTGTTCAAGCATTGAAGTGAACAACAATTGTGAATTGCTTCAAGATCAAGTAGGTGATCATGAATCTGTGGACC
CGTTATTGGATTTTGGATTTGGATTTCCACATGATCATGAAAATGGTCTTAATAACTGCCCTGACAAAGTTGGTGATTTTGGTTCAAGT
TTTTGCTTTAGTGAGTGGGTTGATTTTAGTCATGCTGACATTAAGCCATATTGA

Description of 10 genes surrounding SSR marker TA46 in the order as occur on chromosome Ca4 (cv. Frontier sequence)

(1) Name: Ca_09195
Description: uncharacterized mitochondrial protein AtMg00810-like
Position: Ca4:45,311,344-45,313,485 (- strand)
Length: 2,142 bp

(2) Name: Ca_09196
Description: Protein of Unknown Function (DUF239)
Position: Ca4:45,320,104-45,323,564 (+ strand)
Length: 3,461 bp

(3) Name: Ca_09197
Description: Early Flowering protein
Position: Ca4:45,323,924-45,328,707 (- strand)
Length: 4,784 bp

(4) Name: Ca_09198
Description: 3'-5' exonuclease domain-containing protein
Position: Ca4:45,335,299-45,340,499 (+ strand)
Length: 5,201 bp

(5) Name: Ca_09199
Description: Clathrin interactor EPSIN 1
Position: Ca4:45,342,541-45,348,096 (- strand)
Length: 5,556 bp

(6) Name: Ca_09200
Description: MYB transcription factor MYB185
Position: Ca4:45,365,300-45,368,201 (- strand)
Length: 2,902 bp

(7) Name: Ca_09201
Description: Glycosyl hydrolase family 10 protein
Position: Ca4:45,373,127-45,377,126 (+ strand)
Length: 4,000 bp

(8) Name: Ca_09202
Description: Homeobox protein knotted-1
Position: Ca4:45,415,634-45,420,208 (+ strand)
Length: 4,575 bp

(9) Name: Ca_09203
Description: MYB transcription factor MYB92
Position: Ca4:45,444,888-45,446,396 (+ strand)
Length: 1,509 bp

(10) Name: Ca_09204
Description: uncharacterized protein LOC100797104
Position: Ca4:45,455,150-45,457,034 (+ strand)
Length: 1,885 bp
